# Orbital metastasis from breast carcinoma: structured literature review and report of four cases

**DOI:** 10.3389/fopht.2026.1749352

**Published:** 2026-06-19

**Authors:** Christian Kim, Mica Bergman, Roxana Fu, Caroline Vargason, Michael A. Burnstine

**Affiliations:** 1 Department of Ophthalmology, George Washington University, Washington, DC, United States; 2 Samsun Clinic, Santa Barbara, CA, United States; 3 Department of Ophthalmology, New York University (NYU) Langone Health’s, New York, NY, United States; 4 NAVA Face and Eye, Nampa, ID, United States; 5 Department of Ophthalmology, University of Southern California (USC) Roski Eye Institute, Los Angeles, CA, United States; 6 Eyesthetica, Los Angeles, CA, United States

**Keywords:** breast cancer, breast carcinoma, orbital metastasis, oculoplastics, HER2, estrogen receptor, receptor discordance, FES PET/CT

## Abstract

**Purpose:**

Breast carcinoma is the most commonly diagnosed cancer worldwide and represents the most common primary tumor metastasizing to the orbit. Herein, we present the largest review of the clinical features of orbital metastases from breast carcinoma reported in the literature and describe four additional patients.

**Methods:**

Case reports and case series of biopsy-proven orbital metastases from breast carcinoma published between 2011 and 2025 were identified through a MEDLINE search. Of 220 records screened, 63 articles (29%) met inclusion criteria. Titles, abstracts, and full texts were reviewed for eligibility. Data extracted included patient demographics, tumor subtype and receptor status, timing of breast cancer diagnosis relative to orbital presentation, clinical features, imaging findings, treatments, and survival outcomes.

**Results:**

Sixty-nine cases of breast carcinoma metastatic to the orbit from 63 articles were reviewed. The majority of patients were female (66 of 69, 96%). The average age at presentation of orbital symptoms was 60 years. Orbital metastasis was the presenting manifestation of breast cancer in 30/69 patients (43%). In 15 of 69 patients (22%), orbital metastasis was initially diagnosed as a different orbital process. Among the 49 patients for whom primary tumor type was reported, 25 of 49 (51%) had lobular carcinoma and 21 of 49 (43%) had ductal carcinoma. Two patients had independent lobular and ductal primaries, and one patient had a phyllodes tumor. The most common presenting orbital signs or symptoms were diplopia and/or limited motility (45 of 69, 65%) and decreased or blurry vision (33 of 69, 48%). Treatment of the primary breast carcinoma with orbital metastases varied widely and changed over time with near-equal representation of chemotherapy, hormonal therapy, and radiation therapy. Documentation of survival time was inconsistent.

**Conclusions:**

Breast carcinoma, including metastatic disease, is increasingly characterized by improved survival and prolonged disease control in selected patients with advances in systemic therapy. In patients with a history of breast cancer, metastatic disease should be considered in those presenting with orbital pathology. Conversely, unexplained orbital findings may warrant evaluation for occult malignancy.

## Introduction

Breast carcinoma is the most commonly diagnosed cancer worldwide, with approximately 2.3 million new cases per year (including approximately 250,000 new cases per year in the United States) (1). Only a small percentage of patients with breast cancer (<1%) suffer from orbital metastasis (2). Of all oncology oculofacial patients with orbital metastasis, 1%–2% are metastatic breast cancer (3–5). The manifestations, treatment, and course of orbital metastases vary. In this article, the authors review published case reports of orbital metastases from breast cancer patients between 2011 and 2025 and report four additional patients with orbital breast cancer metastasis.

## Methods

The MEDLINE database was searched using the terms (“breast cancer” OR “breast carcinoma”) AND (orbit OR orbital) AND (metasta)* with a filter for the English literature and publication dates 2011–2025. The date of the last search was 11/17/25. A 15-year time frame was selected to capture contemporary diagnostic and therapeutic approaches. A keyword-based search strategy was employed to maximize sensitivity for case reports and case series, which may not always be consistently indexed using controlled vocabulary. MEDLINE was selected as the primary database given its comprehensive indexing of peer-reviewed biomedical literature and widespread use in ophthalmology and oncology research. Given the focused scope on biopsy-proven orbital metastases from breast carcinoma, MEDLINE was deemed sufficient to capture the majority of relevant case reports.

The inclusion criteria comprised case reports or case series published in English between 2011 and 2025 that specifically described orbital metastasis from breast carcinoma, with diagnosis confirmed by biopsy. All age groups were included. Exclusion criteria included reports of non-orbital metastases, primary orbital tumors, and cases lacking definitive diagnostic confirmation.

This search yielded 220 articles, all of which were screened for relevance. Titles and abstracts were screened for relevance, followed by full-text review to confirm eligibility. Screening was performed by the authors. Case reports of biopsy-proven orbital metastases with a breast cancer primary were included. Data collected, when available, included patient demographics, breast cancer type and receptor status, knowledge of breast cancer at time of orbital presentation, types of orbital symptoms, diagnostic imaging, systemic and orbital treatments, clinical course, and survival time.

**Figure 1:**
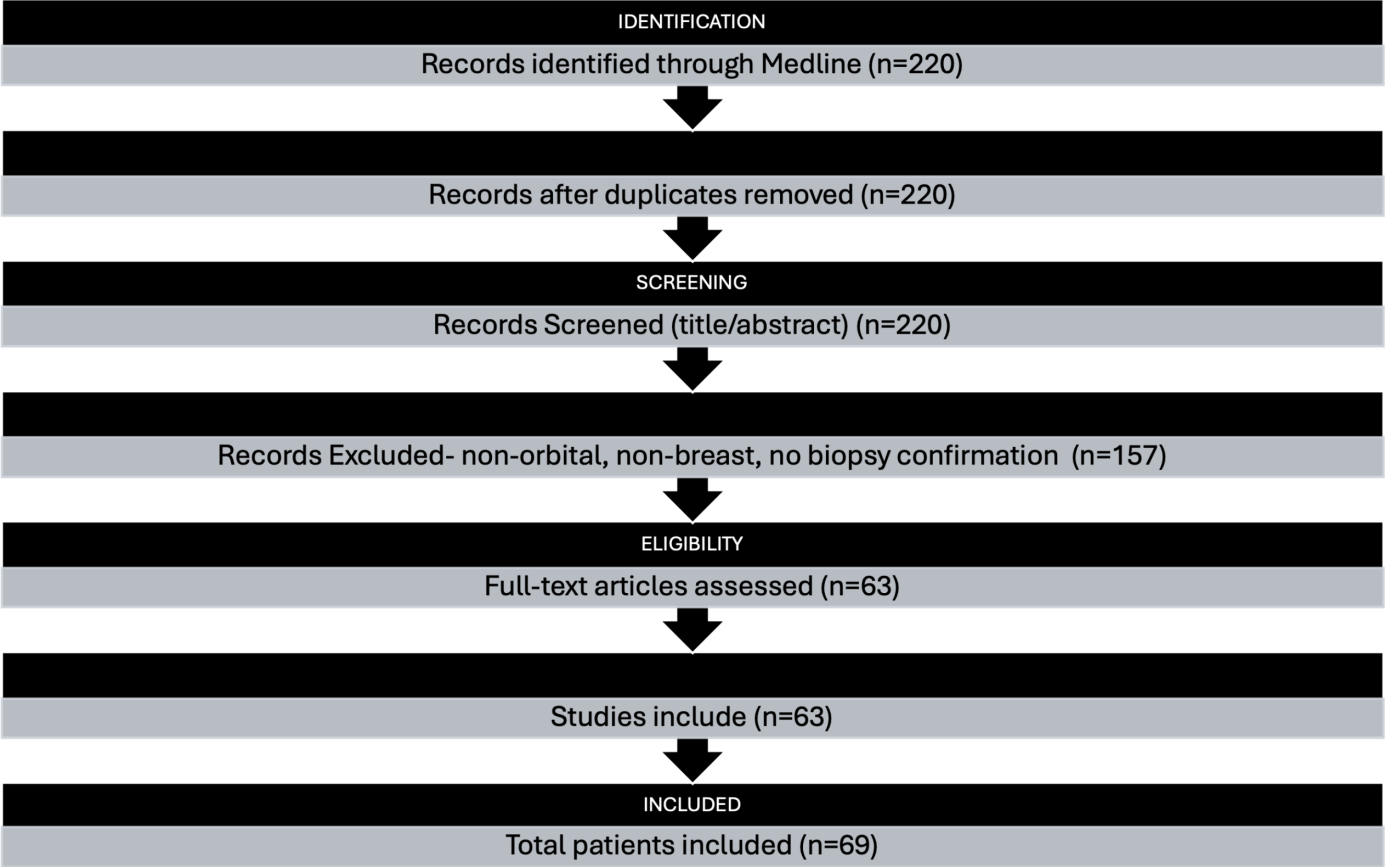
Literature search of the MEDLINE database (2011–2025) identified 220 records. After title and abstract screening, non-relevant articles were excluded, and full-text review was performed on remaining studies. Articles were excluded if they did not report orbital metastasis from breast carcinoma, lacked biopsy confirmation, or described primary orbital tumors or non-orbital metastases. A total of 63 articles met inclusion criteria, comprising 69 cases of biopsy-proven orbital metastasis from breast carcinoma included in this review.

The four additional cases presented in this manuscript were contributed by the co-authors. A PRISMA-style flow diagram was constructed to illustrate the study selection process (**Figure 1**). Sixty-nine cases of breast carcinoma metastatic to the orbit from 63 articles were included. We acknowledge that limiting the search to a single database may have resulted in the omission of additional relevant cases indexed elsewhere, which represents a limitation of this review.

## Results

### Literature review

Sixty-nine cases of breast carcinoma metastatic to the orbit from 63 articles were included. The 63 included articles represented 29% of the 220 screened records (3–53, 57–67). There were 66 females (66 of 69, 96%) and 3 males (3 of 69, 4%). Average age at first presentation of orbital symptoms was 60 years (range: 31–83, median = 62). Of the 69 patients, 39 (57%) had a known diagnosis of breast cancer, whereas in 30 patients (43%), orbital metastasis was the initial presentation. For those with a known breast cancer diagnosis, the average time from diagnosis to orbital metastasis was 7.7 years (range: 0–35, median = 7). The average age at presentation was similar whether or not the patient had a known breast cancer diagnosis (mean = 59 and 61, respectively; median = 62 and 64.5, respectively).

Among the 49 patients for whom primary tumor type was reported, 25 of 49 (51%) had lobular carcinoma, 21 of 49 (43%) had ductal carcinoma, and 1 of 49 (2%) had a phyllodes tumor. Two of the 49 patients had independent lobular and ductal primary tumors. In 20 of 69 patients (29%), the primary tumor type was unknown or not reported. Orbital metastasis was right-sided in 30 of 69 patients (43%), left-sided in 26 of 69 (38%), and bilateral in 13 of 69 (19%). Patients with bilateral orbital metastases presented at a similar age as those with unilateral presentations (mean = 61 and 60, respectively; median = 62 and 60, respectively).

Of the 69 patients with biopsy-proven orbital metastases, 67 underwent surgical biopsy, and 3 underwent fine needle aspiration biopsy. The sum exceeds the total number of patients, as one patient had an inconclusive fine needle aspiration biopsy with a subsequent conclusive surgical biopsy.

Of the 69 cases, the most common orbital sign or symptom was diplopia and/or limited motility, which was seen in 45 of 69 patients (65%). Decreased visual acuity and/or blurry vision was seen in 33 of 69 patients (48%). Among the 33 patients with decreased visual acuity and/or blurry vision, 6 of 33 (18%) had a relative afferent pupillary defect, and 5 of 33 (15%) had a central scotoma visual field defect; three patients had both findings. Ptosis was seen in 24 of 69 patients (35%) and lagophthalmos in 5 of 69 (7%). Exophthalmos was noted in 22 of 69 patients (32%) and enophthalmos in 10 of 69 (14%). Pain or headache was present in 19 of 69 patients (28%), edema and/or erythema in 21 of 69 (30%), and a palpable mass in 13 of 69 (19%). Anterior segment findings, such as conjunctival hyperemia, were reported in 15 of 69 patients (22%), and posterior segment findings, including blurred disc margins or optic disc edema, were reported in 4 of 69 (6%). Other findings, such as tearing or hypesthesia, were reported in 8 of 69 patients (12%).

Imaging studies were described in all but one case. Metastases varied widely in orbital location, ranging from the orbital apex posteriorly to the palpebral tissue anteriorly. Lesions were found in both intraconal and extraconal locations. In some cases, the lesions were discrete, whereas in others they were infiltrative. Extraocular muscle involvement was reported in at least 36 cases; however, the total number of cases in which this variable was explicitly assessed was not consistently reported across studies. Similarly, bony involvement was described in 11 cases, but the denominator of cases reporting this variable could not be reliably determined. Of the 21 ductal carcinomas, 6 (29%) showed bony involvement and 14 (66%) showed muscle involvement. Of the 25 lobular carcinomas, 5 (20%) showed bony involvement and 17 (68%) showed muscle involvement. The orbital metastasis in the patient with both ductal and lobular carcinoma showed muscle involvement.

In 15 of 69 patients (22%), the orbital metastasis was initially diagnosed as a different orbital process. Misdiagnoses included non-specific orbital inflammation (6 of 15, 40%), sinusitis (3 of 15, 20%), cellulitis (3 of 15, 20%), IgG4-related disease (1 of 15, 7%), sarcoidosis (1 of 15, 7%), and lymphoma (1 of 15, 7%).

Treatment of the primary breast cancer and the orbital metastases varied widely depending on the nature and molecular profiles of the primary tumors, the nature and location of the orbital tumors, and the presence or absence of lymph node spread and other metastases. Treatment data following identification of orbital metastasis were available for 58 of 69 patients (84%). Among these 58 patients, hormone therapy was used in 33 of 58 (57%), chemotherapy in 34 of 58 (59%), and radiation therapy in 26 of 58 (45%). Multimodal treatment was the norm. Among the 58 patients with treatment data, 8 of 58 (14%) received hormone therapy alone, 8 of 58 (14%) chemotherapy alone, and 3 of 58 (5%) radiation therapy alone; 7 of 58 (12%) received all three modalities. The extent of tumor debulking or resection during biopsy, if performed, was not consistently documented. Documentation of outcome measures and survival time was inconsistent and did not allow for meaningful analysis.

### Case reports

#### Case report 1

A 73-year-old female with a history of infiltrating lobular carcinoma estrogen receptor (ER) positive, progesterone receptor (PR) positive, and human epidermal growth factor receptor (HER2) equivocal, diagnosed 10 years prior, and with known metastases to the periaortic lymph nodes, right hip, and cervical spine presented to an oculoplastics clinic with right lower eyelid swelling. On examination, there was a palpable, hard, non-tender, rubbery mass above the right inferior orbital rim. There was no malposition of the right lower eyelid, and no proptosis, globe displacement, or motility disturbance. Best corrected visual acuity was 20/25 in each eye. There was no relative afferent pupillary defect, intraocular pressures were normal, and there were no significant anterior or posterior segment findings. CT demonstrated an inferior orbital mass measuring 2.7 cm × 2 cm. Orbital biopsy was performed and demonstrated metastatic breast carcinoma. The patient underwent orbital radiation but succumbed to her disease 1 year later. There was no known clinical or radiographic follow-up.

#### Case report 2

A 41-year-old female with a history of invasive ductal carcinoma with lobular features (ER negative, PR negative, HER2 negative) diagnosed 12 years prior and recurrence six years prior with bone and lymph node involvement presented to an oculoplastics department with a chief complaint of right upper eyelid heaviness. On examination, she was found to have right-sided enophthalmos and limitation of right eye supraduction. MRI demonstrated infiltration of the intraconal fat and intraconal soft tissues. An orbital biopsy was performed and demonstrated metastatic breast carcinoma. The patient was treated with right orbital radiation and abemaciclib. Two and a half years later, there was interval resolution of the infiltrative mass on MRI. The patient experienced skin and liver metastases one year after orbital metastasis but is currently doing well on capecitabine.

#### Case report 3

A 68-year-old female with a history of invasive ductal carcinoma with mucinous and papillary features (ER positive, PR positive, HER2 negative) diagnosed 6 years ago with known metastases to the bones, adrenal gland, liver, omentum, and subcutaneous tissues of the right mid-back, presented to an oculoplastics clinic with diplopia. Best corrected visual acuity was 20/40 on the right and 20/20 on the left. Pupils and intraocular pressures were normal bilaterally. Examination on the right was significant for a palpable mass inferolaterally, which induced a mechanical lateral lower eyelid ectropion. There were dilated conjunctival vessels in the lateral inferior fornix and 1+ conjunctival hyperemia. The patient had 1 mm of a right-sided hyperglobus, along with right-sided infraduction deficit. CT demonstrated an inferolateral orbital lesion measuring 2.2 cm × 1.6 cm × 1.7 cm. An orbital biopsy was performed and demonstrated metastatic breast carcinoma. Shortly thereafter, additional metastases were discovered involving the thoracic and lumbar spines, multiple ribs, the right adrenal gland, the liver, the omentum, and the subcutaneous tissues of the back. Systemically, the patient was started on palbociclib and Faslodex. Progression of liver and omental metastases was noted five months later, and capecitabine was initiated. The patient’s orbit was treated with gamma knife radiotherapy. An MRI 7 months post-operatively showed a decrease in orbital tumor burden. Clinically, the patient showed resolution of diplopia without abduction deficits.

The patient was subsequently treated with abemaciclib and exemestane (Aromasin), which she self-discontinued due to poor tolerance. Significant progression of metastatic disease in the right adrenal gland, lymph nodes, and extensive bony metastases was noted ten months later. In the setting of diffuse metastatic disease and limited treatment tolerance, the patient elected to forgo further systemic or radiation therapy and enrolled in hospice care.

#### Case report 4

**Figure 2:**
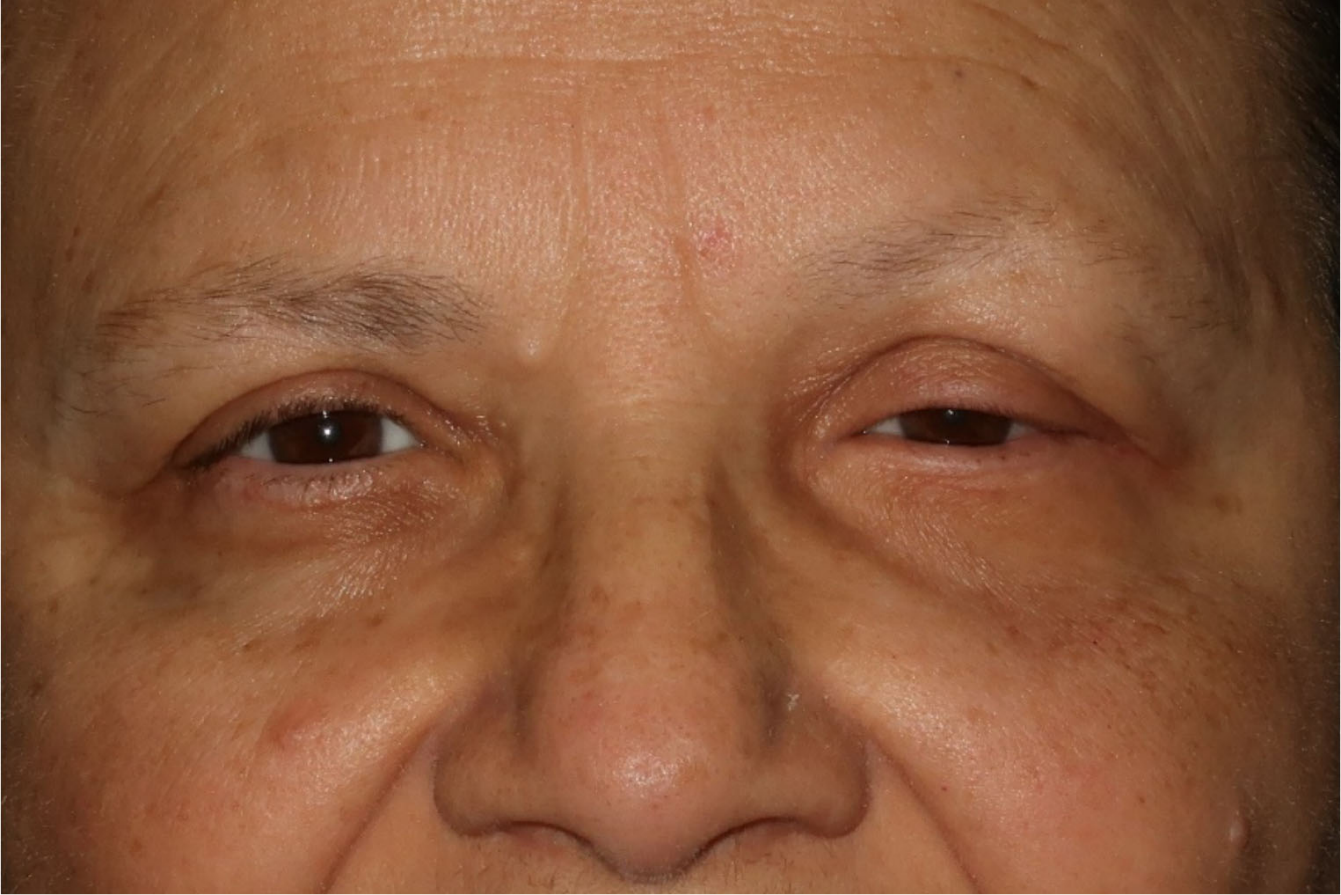
63-year-old woman with 1mm enophthalmos and 1.5mm of left upper eyelid ptosis due to infiltrating lobular breast carcinoma.

A 63-year-old female was referred for a 9-month history of left upper eyelid ptosis with periorbital swelling (**Figure 2**). She had no past ocular history. Past medical and surgical history was notable for a history of intraductal breast carcinoma diagnosed and managed 2 years ago with a modified radical mastectomy (ER/PR positive; HER2 negative by IHC and FISH; 28/33 positive lymph nodes). She had a history of hypertension, hypercholesterolemia, and diabetes. Visual acuity without correction was 20/30 OD and 20/20 OS. She had no afferent pupillary defect, and confrontation, and extraocular motility exams were normal. She had 1.5 mm of left upper eyelid ptosis with an increase in her margin to fold distance (MFD), 1 mm of left-sided enophthalmos, and firmness in the left upper and lower orbit. Slit lamp, intraocular pressure, and dilated funduscopic examinations were normal. A driver’s license photograph from 5 years prior did not demonstrate left upper eyelid ptosis.

A CT scan with IV contrast revealed diffuse left intraorbital enhancement. Orbital biopsies of the superior orbit, lacrimal gland, and inferior orbit were all positive for metastatic lobular carcinoma. Immunostains of tumor cells were positive for CAM 5.2, GATA3, HER2, and PR positive (Dako clone 1294). Stains were negative for p40, TTF-1, CDX-2, and E-cadherin. Notably, this represents receptor discordance from the primary tumor, which was HER2 negative, whereas the metastatic orbital lesion demonstrated HER2 positivity, consistent with receptor conversion.

The bone scan was negative. MRI of the brain and orbits and PET/CT with Cerianna (^18^F-fluoroestradiol, FES) demonstrated uptake in the left orbit, highlighting estrogen receptor–positive metastatic disease and aiding in treatment selection. FES PET/CT (Cerianna) is a targeted imaging modality that allows *in-vivo* assessment of estrogen receptor expression and has emerged as a valuable tool in detecting and characterizing ER-positive metastatic breast cancer, particularly in cases of receptor discordance (54). In this case, it provided functional confirmation of ER-positive orbital metastasis and supported systemic treatment selection. The patient underwent orbital radiation and was started on Kisqali (ribociclib) for hormone receptor–positive disease and Faslodex (fulvestrant) for ER-positive metastatic breast cancer in the setting of receptor discordance. At the 2-year follow-up, her eyelid and orbital examination remain stable, with quiescent disease on serial imaging, and she continues annual surveillance with FES PET/CT (Cerianna) scans.

## Discussion

Orbital metastases represent approximately 3.5%–7% of orbital tumors (55, 56). Of these, breast cancer is the most common secondary/metastatic tumor (55, 57, 58). Clarifying the nature of breast cancer metastases to the orbit facilitates our ability to provide earlier diagnosis and tailored management. We present the largest case series to date and suggest that, with contemporary treatment regimens, metastatic breast carcinoma may increasingly be managed as a chronic disease rather than an immediate death sentence.

The vast majority of breast cancers are epithelial carcinomas. Of these, invasive ductal carcinomas represent the vast majority (50%–80% of all breast cancers), and invasive lobular carcinomas are the second-most prevalent (5%–15% of all breast cancers) (59). Among the 49 patients for whom primary tumor type was reported, lobular carcinoma accounted for 25 of 49 cases (51%) and ductal carcinoma for 21 of 49 cases (43%). This breakdown is similar to that seen by Sindoni et al. (50% lobular, 50% ductal) (60), and Blohmer et al. (32% lobular, 50% ductal) (61), but varies significantly from the companion reports by Jakobiec et al. and Homer et al. (100% lobular) (62, 63) and the findings by Raap et al. (88% lobular, 12% ductal) (15). Jakobiec et al. propose that the over-representation of invasive lobular metastases in the orbit is a result of estrogen receptor positivity in a high estrogen setting (62, 64) along with the absence of e-cadherin, which normally limits dispersion, thus facilitating stromal dispersion in the foreign tissue (62). The reason for the discrepancy between lobular predominance versus lobular and ductal parity among these studies is unclear and should be the subject of further investigation. Additionally, contemporary series highlight that biomarker and receptor status may differ between the primary tumor and orbital metastases, underscoring the importance of biopsy and repeat molecular profiling to guide treatment decisions (65).

The most common orbital signs and symptoms in this series were diplopia/limited motility (45 of 69, 65%), decreased or blurry vision (33 of 69, 48%), ptosis (24 of 69, 35%), edema/erythema (21 of 69, 30%), pain/headache (19 of 69, 28%), and enophthalmos (10 of 69, 14%) (**Table 1**) (60, 61, 66, 67).

**Table 1:** Common presenting signs and symptoms among patients with orbital metastases from breast carcinoma (*n* = 69).

Sign/Symptom	Frequency, n/N (%)
Diplopia/limited motility	45/69 (65%)
Decreased/blurry vision	33/69 (48%)
Ptosis	24/69 (35%)
Edema/erythema	21/69 (30%)
Pain/headache	19/69 (28%)
Enophthalmos	10/69 (14%)

We investigated the relationships between particular signs/symptoms and breast cancer type. Among the 21 patients with ductal carcinoma metastases, 8 of 21 (38%) presented with exophthalmos and 1 of 21 (5%) with enophthalmos. Among the 25 patients with lobular carcinoma metastases, 5 of 25 (20%) presented with exophthalmos and 5 of 25 (20%) with enophthalmos. This discrepancy may be explained by the proclivity for stromal dispersion observed in invasive lobular carcinoma (62). Recent data from Peschiaroli et al. further characterize orbital metastases from breast cancer, noting a predilection for the upper lateral extraconal space (approximately 50% of cases), with intraconal involvement in 30% and bilateral disease in up to 20% of patients. These findings support the heterogeneous clinical presentations observed in our cohort and reinforce the importance of maintaining a broad differential diagnosis (65).

Certain signs and symptoms were similarly represented in ductal and lobular carcinomas, whereas other signs and symptoms were far more common in one subtype than the other. Among the 21 patients with ductal carcinoma metastases, 14 of 21 (67%) presented with motility disturbance or diplopia, compared with 14 of 25 (56%) of patients with lobular carcinoma metastases. Similarly, pain was reported in 7 of 21 ductal metastases (33%) and 7 of 25 lobular metastases (28%). Vision loss was more common in ductal carcinoma metastases (12 of 21, 57%) than in lobular metastases (9 of 25, 36%). Edema or erythema was more common in lobular carcinoma metastases (10 of 25, 40%) than in ductal metastases (5 of 21, 24%), as was a palpable mass (8 of 25, 32% vs. 2 of 21, 10%). The nature of the study precluded statistical analyses of relationships between variables. The reasons for these differences are unknown and should be the subject of future studies.

Male breast cancers account for about 1% of all breast cancer cases and typically show patterns consistent with, but sometimes slightly different from, their female counterparts (68, 69). This study included three patients with male breast cancer with orbital metastases. All three patients presented within one standard deviation of the mean presenting age. Two patients already carried a breast cancer diagnosis. The primary tumor was a ductal carcinoma in one patient, a lobular carcinoma in another, and unknown in the third.

In 30 of 69 patients (43%), orbital metastasis represented the first presentation of breast cancer, a higher proportion than that seen in other studies (2). Series that draw on a single institution may more accurately reflect the true proportion of novel presentations as published case reports, particularly when the field is more mature, are more likely to represent unusual presentations. Recognizing the possibility of occult malignancy remains paramount.

In this series, 15 of 69 patients (22%) were initially misdiagnosed. Among these 15 patients, 6 of 15 (40%) received an infectious diagnosis (sinusitis or cellulitis), 8 of 15 (53%) received an inflammatory diagnosis (non-specific orbital inflammation, sarcoidosis, or IgG4-related disease), and 1 of 15 (7%) received a diagnosis of lymphoma. Inflammatory misdiagnoses occurred in cases where there was no existing breast cancer diagnosis, and infectious misdiagnoses occurred in cases where there was an existing breast cancer diagnosis. This result highlights the importance of appreciating that orbital infiltration or an orbital mass may represent breast cancer metastasis.

Much like orbital malignancies may be misdiagnosed as other entities, so too can other orbital processes be misdiagnosed as neoplasms. Orbital masses in patients with a history of breast cancer are often presumed to represent metastasis, but at times, an alternate etiology is discovered. For example, Murchison et al. describe an 82-year-old female with a history of scirrhous breast cancer presenting with diplopia, restricted motility, and enophthalmos. Initially, a diagnosis of metastasis was made, but a biopsy ultimately revealed no malignant cells but the presence of amyloid deposits (70). Similarly, Henderson et al. describe a 74-year-old female with a history of breast cancer who presented with diplopia and left upper eyelid swelling, who was found on imaging to have a mass within the superior rectus-levator complex. While initially worrisome for metastasis, biopsy demonstrated a necrotizing, granulomatous parasitic infection (71). Rajabi et al. describe a 52-year-old female with a breast cancer history who presented with pain and vision loss. She was found to have an intraconal mass that, on biopsy, revealed a blood clot (72). Finally, Bacorn et al. report a female patient with a history of breast cancer, who presented with diplopia and was found on imaging to have a medial rectus mass. Orbital biopsy was expected to reveal metastatic ductal carcinoma consistent with her primary tumor but instead demonstrated a gastrointestinal neuroendocrine tumor. Ultimately, not only was the orbital mass diagnosis revised, but also the original tumor, which was determined to be a metastasis, itself, from the gastrointestinal primary (73).

Radiation therapy is the primary local treatment for orbital metastasis from breast carcinoma, providing effective symptom control and preservation of vision. Recent systematic reviews further support the role of radiotherapy as a safe and effective treatment modality, with most patients experiencing symptomatic improvement and low rates of toxicity, typically limited to mild (grade 1–2) adverse effects such as conjunctivitis, dermatitis, or xerophthalmia (74). Most patients receive external beam radiation in palliative doses, typically 30 Gray in 10 fractions, with high rates of improvement in orbital symptoms (75). Radiation is particularly indicated for symptomatic lesions or when local disease control is required. Advanced radiation techniques, including intensity-modulated radiation therapy (IMRT) and stereotactic body radiotherapy (SBRT), may allow for improved target conformality and sparing of adjacent critical structures, particularly in anatomically complex orbital lesions (74). Imaging typically demonstrates diffuse orbital fat infiltration with hypointense signal on both T1- and T2-weighted sequences and marked contrast enhancement, often with associated extraocular muscle involvement, reflecting the infiltrative nature of these lesions (65). While surgery is rarely performed, it may be considered in select situations for diagnostic clarification or palliative purposes (75).

Systemic and biologic therapies are selected according to breast cancer subtype and prior treatment history. In hormone receptor–positive, human epidermal growth factor receptor 2 (HER2)–negative disease, endocrine therapy (such as aromatase inhibitors or fulvestrant) is combined with cyclin-dependent kinase 4 and 6 (CDK4/6) inhibitors, including palbociclib (35, 76). Chemotherapy, including capecitabine, is generally reserved for endocrine-resistant cases. In HER2-positive disease, anti-HER2 agents such as trastuzumab, pertuzumab, and trastuzumab emtansine are used in combination with taxane chemotherapy (77, 78). For patients with central nervous system involvement, small-molecule HER2 inhibitors like tucatinib and lapatinib are preferred due to enhanced CNS penetration (77). In triple-negative breast cancer, single-agent chemotherapy is standard, with the addition of immunotherapy for programmed death-ligand 1 (PD-L1)–positive tumors or poly(ADP-ribose) polymerase (PARP) inhibitors for germline BRCA-mutated cases (79). Biologic agents remain integral across subtypes, with durable responses reported when combined with systemic therapy and radiation. A multidisciplinary approach is essential, with treatment individualized based on tumor biology, extent of systemic disease, and patient treatment response status (79).

This study has limitations. First, the data is drawn from a wide array of clinicians and institutions. While this does confer meaningful benefits of increasing quantity and reducing bias, it introduces variability into the assessment, management, and documentation of each patient, which may limit the reliability of cross-comparisons and the generalizability of results. Second, when assessing patient findings, subjective and objective findings were combined. For example, diplopia and motility limitations were categorized together, as were blurry vision and decreased vision, among others. These symptoms and signs should and often do co-vary. Reliance in some cases on subjective measures limits the reliability of the data. Finally, the authors of each case report varied reporting, such that a complete dataset was not available for each patient.

To conclude, in patients with a history of breast cancer, metastatic disease should be a consideration in any patient with orbital pathology. Orbital pathology without clear etiology should raise suspicion for occult malignancy. Metastatic breast cancer should no longer be considered a death sentence and may be managed by an oncologist with multiple modalities.

## Data Availability

The datasets for this article are not publicly available due to concerns regarding participant/patient anonymity. Requests to access the datasets should be directed to the corresponding authors.
